# Chronic Kidney Disease Presenting With Brown Tumors in the Mandible

**DOI:** 10.7759/cureus.23985

**Published:** 2022-04-09

**Authors:** Sumedha Singh, Santosh K Padhy, Satya S Mohapatra, Adya Panda, Pratyush Shahi

**Affiliations:** 1 Radiology, Institute of Medical Sciences and SUM Hospital, Bhubaneswar, IND; 2 Radiodiagnosis, Institute of Medical Sciences and SUM Hospital, Bhubaneswar, IND; 3 Orthopaedics, University College of Medical Sciences, Delhi, IND

**Keywords:** tertiary hyperparathyroidism, chronic kidney disease, secondary hyperparathyroidism, multiple, mandible, brown tumor

## Abstract

A 45-year-old man with stage 4 chronic kidney disease (CKD) on controlled dialysis presented with right-sided painful jaw swelling and protruding into the oral cavity for one year. Examination revealed a 3 x 2.5-cm hard, fixed, and tender swelling of the right mandible. Imaging showed expansile radiolucent lesions in bilateral retromolar regions of the mandible, local destruction of the basal bone, and diffuse osteopenia of the skull. Laboratory investigations revealed elevated parathyroid hormone (PTH), elevated serum calcium, normal serum phosphorous, and elevated alkaline phosphatase (ALP). A provisional diagnosis of tertiary hyperparathyroidism (HPT) causing brown tumors was made, which was confirmed on histopathology. Surgical removal of the lesion and subtotal parathyroidectomy were done followed by cinacalcet and controlled dialysis. This case report highlights the possibility of encountering multiple focal brown tumors in a patient and the importance of their differentiation from malignancy.

## Introduction

Chronic kidney disease (CKD) often results in osteodystrophy due to secondary or tertiary hyperparathyroidism (HPT). Elevated parathyroid hormone (PTH) causes osteoclastic resorption of the bones that leads to various skeletal manifestations, one of which is a brown tumor. Multiple brown tumors are rare and can be easily mistaken for malignancy [1]. We present such a case of CKD with bilateral lesions in the mandible, one of them large enough to protrude into the oral cavity and cause symptoms, and highlight the importance of differentiating them from malignancy.

## Case presentation

A 45-year-old man presented with right-sided painful jaw swelling and protruding into the oral cavity for one year. It had an insidious onset and gradual progression to its present size. Chewing exacerbated the associated pain. The patient had known comorbidities of hypertension and stage 4 CKD and had been undergoing dialysis three times weekly for nine years.

Examination revealed a 3 x 2.5-cm hard, fixed, tender swelling of the right mandible protruding into the oral cavity. The overlying mucosa was intact. There was an associated loss of the right third molar tooth with bleeding sites on its fragile mucosa.

**Figure 1:**
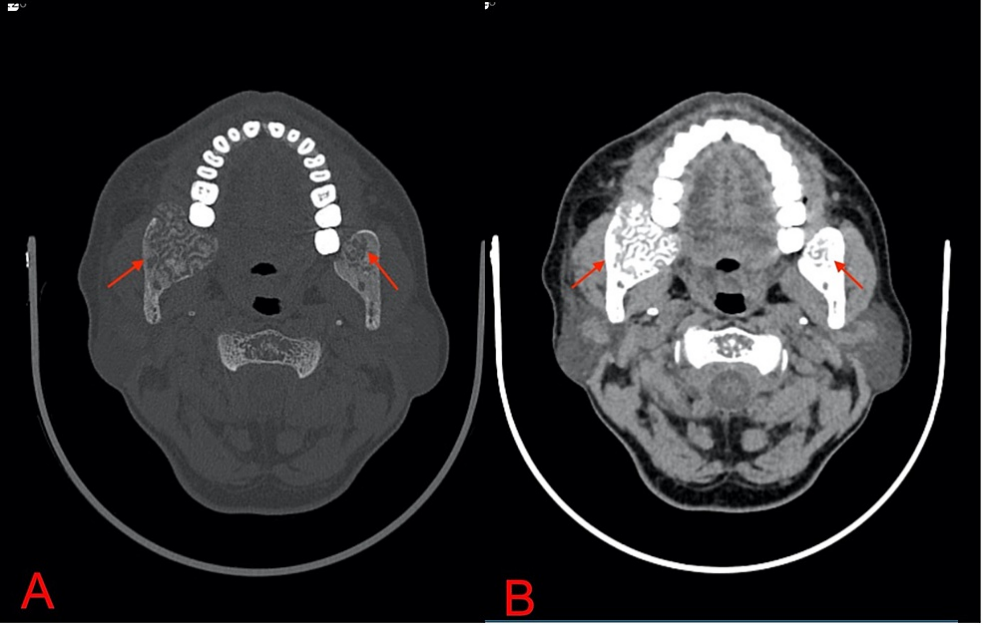
Axial CT [bone window (A) and soft tissue window (B)] The images show expansile radiolucent lesions with linear and branching patterns of resorption in bilateral retromolar regions of the mandible (right>left), causing local destruction of the basal bone under the apices of bilateral molar teeth. Loss of the right third molar tooth is seen, likely due to the lesion CT: computed tomography

**Figure 2:**
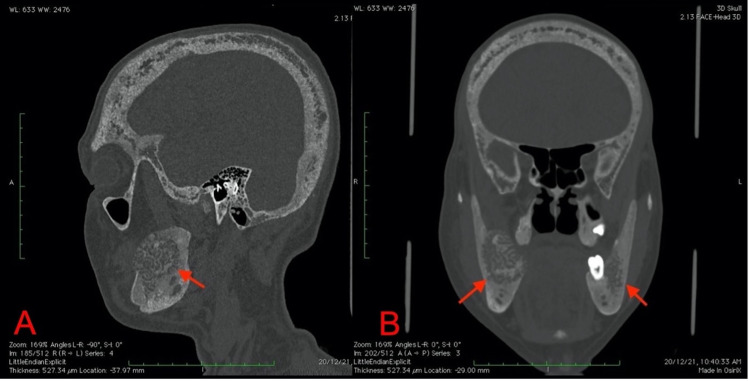
Sagittal (A) and coronal (B) CT in the bone window show the lesions with associated diffuse osteopenia of the skull CT: computed tomography

**Figure 3:**
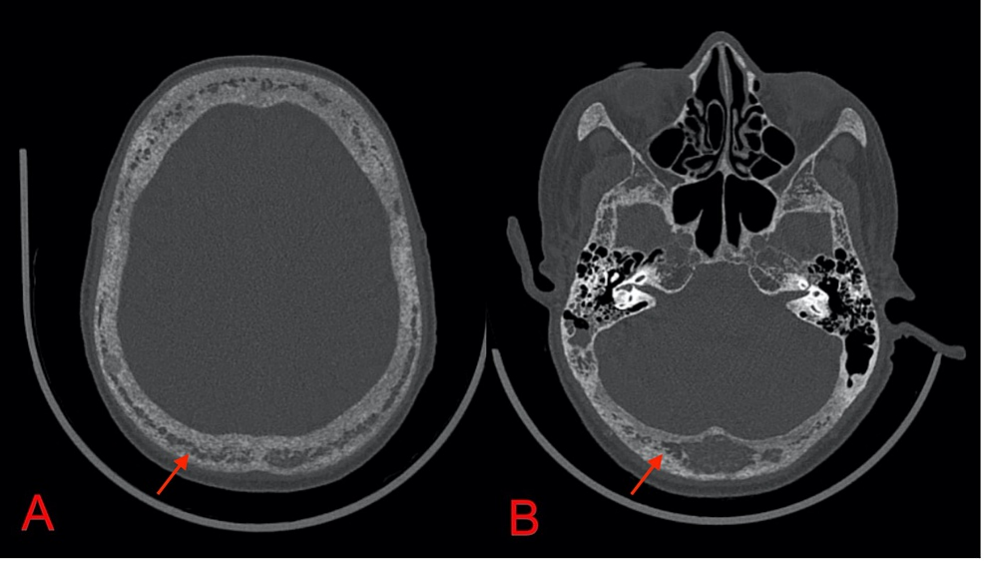
CT scans of the calvaria (A) and skull base (B) in the bone window show diffuse spotty osteopenia with salt and pepper morphology CT: computed tomography

Laboratory investigations revealed elevated PTH levels of 2,933 pg/dl [target range for stage 4 CKD as per Kidney Disease Improving Global Outcomes (KDIGO) guidelines: 70-110 pg/dl], elevated serum calcium of 13 mg/dl (normal range, 8.8-11 mg/dl), elevated alkaline phosphatase (ALP) of 1,758 IU/l (normal range, 65-300 IU/l), and elevated serum phosphorus of 5.5 mg/dl (normal range: 2.5-5.0 mg/dl), indicating uncontrolled severe tertiary HPT. CT scan revealed expansile radiolucent lesions in bilateral retromolar regions of the mandible, local destruction of the basal bone, and diffuse osteopenia of the skull (Figures 1-3).

A provisional diagnosis of brown tumor was made, which was confirmed on histopathology. Total surgical removal of the lesion on the right side was done with curettage and extraction of the element involved. However, the lesion in the left hemimandible was kept under observation due to its small size. Subtotal parathyroidectomy was also undertaken, leaving 40 mg of residual parathyroid tissue of the left inferior gland. Additionally, cinacalcet 30 mg and controlled dialysis were continued with periodic monitoring of serum calcium and phosphorus to evaluate the response. Cinacalcet was titrated every two to four weeks through sequential dosing of 30, 60, 90, 120, and 180 mg once daily to target the PTH levels of 70 to 110 pg/mL. Serum calcium and phosphorus were measured within one week and PTH was measured at two weeks followed by four weeks after the initiation of the dose, which reflected a resolving trend. At the one-year follow-up, there were no signs of recurrence on the right side with a mild degree of resolution of the left mandibular lesion. The patient showed a significant improvement in his overall well-being.

## Discussion

HPT is a state of excess serum levels of PTH that can be due to primary, secondary, or tertiary causes. Primary HPT can be due to parathyroid adenoma (85% of cases), hyperplasia (15% of cases), or carcinoma (<1% of cases) and is characterized by hypercalcemia and hypophosphatemia. Secondary HPT can be caused by CKD, malnutrition, or vitamin D deficiency and, contrary to primary HPT, is characterized by decreased serum calcium and normal or increased serum phosphorous, whereas tertiary HPT is characterized by hypercalcemia and hyperphosphatemia [2].

CKD results in skeletal manifestations such as osteomalacia in adults and rickets in children due to secondary HPT. These are collectively referred to as renal osteodystrophy. Radiographs can also show a diffuse increase in bone radiodensity, seen more often in the axial skeleton. The etiopathogenesis of this diffuse osteosclerosis is not well understood, although it can reflect the anabolic effect of PTH. Despite the increased radiodensity, the bone is structurally weak with increased chances of stress fracture [3]. The spine demonstrates alternating hyperdense endplates and hypodense vertebral body center, giving a characteristic striped appearance, which is also known as the rugger-jersey spine.

**Figure 4:**
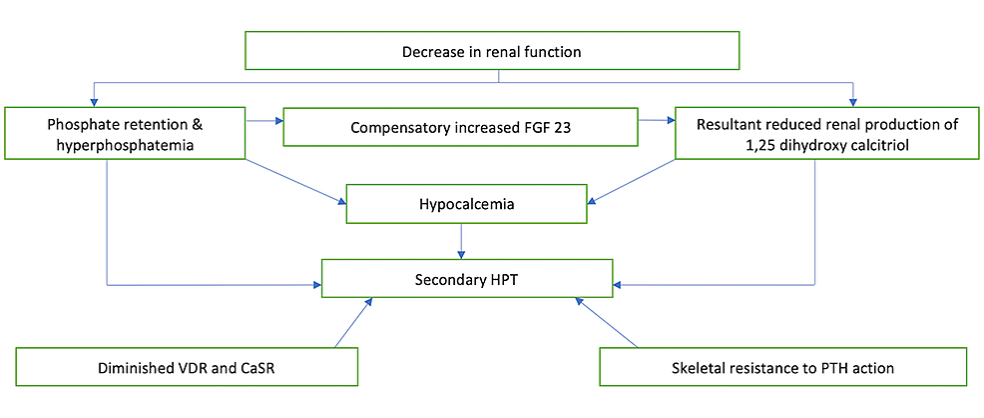
Etiopathogenesis of secondary hyperparathyroidism (HPT) FGF 23: fibroblast growth factor 23; VDR: vitamin D receptor; CaSR: calcium-sensing receptor; PTH: parathyroid hormone

One of the late manifestations of the condition is a brown tumor (osteitis fibrosa cystica). It gets its name from the brown color on histology due to hemosiderin deposition. Brown tumor formation occurs in CKD due to secondary HPT, which has a multifactorial etiopathogenesis. A decrease in renal function causes phosphate retention, which triggers the release of fibroblast growth factor 23 (FGF 23) from the osteocytes. FGF 23 decreases the renal production of 1,25 dihydroxy calcitriol, thereby stimulating the release of PTH. Diminution in the expression of vitamin D receptor (VDR) and calcium-sensing receptor (CaSR) in parathyroid cells and skeletal resistance against PTH in patients with CKD further contributes to the hypocalcemia-secondary HPT cascade [2]. Tertiary HPT entails the development of autonomous parathyroid action due to long-standing secondary HPT in CKD. The etiopathogenesis is summarized in Figure 4.

Brown tumor is commonly found in the pelvis, ribs, femur, and facial bones [4]. Clinically, brown tumors classically present with features of secondary HPT referred to as “bone, stone, abdominal groan, and psychic moan”. The factors responsible for these symptoms are hypercalcemia, excessive resorption of bone, and mental agony due to the same. It mostly affects women in the fourth to fifth decades of life [5]. Radiologically, when involving the mandible, it appears as a unilocular or multilocular lucency, which can be associated with bony expansion, deformity, tooth mobility, and loss of lamina dura [6]. The cortex of the mandible appears thinned out due to demineralization, and the inferior border, cortical outlines of maxillary sinuses, and mandibular canal are often involved. Due to the reduced density of the mandible, teeth often stand out in contrast [5]. A diagnostic confusion arises when there are multiple lytic lesions involving different areas of the skeleton, which may mimic metastatic disease [6].

The best method for diagnosing brown tumors is a parathyroid immunoassay. Further workup with bone scintigraphy (using technetium 99m-methylene diphosphonate) can be done, which shows intense uptake by brown tumor and helps in localizing other areas of similar activity [7].

The treatment of brown tumors is mainly pharmacological and is done by correcting the underlying HPT. In cases of tertiary HPT, it is treated by correcting HPT with calcimimetics, and controlled dialysis, renal transplant, or subtotal parathyroidectomy may also be needed. Surgical excision of the tumor is sometimes necessary, like in our case as it was protruding into the oral cavity and causing symptoms [8]. Some advocate curettage with wound packing, allowing for secondary healing, in addition to the adjunctive treatment of the underlying disease [4].

## Conclusions

Multiple brown tumors can be seen in the mandible in patients with CKD as a result of secondary or tertiary HPT. Although they can mimic malignancy on imaging, reaching the correct diagnosis is possible with appropriate clinical history and biochemical tests. Early diagnosis and treatment lead to an overall improvement of the prognosis. Surgical removal of the lesion may be required in case of symptoms.
